# Christoph Cantzler, Sigrid Harendza: Gute Besserung – Das Krankenhausspiel

**DOI:** 10.3205/zma001430

**Published:** 2021-02-15

**Authors:** Götz Fabry

**Affiliations:** 1 Albert-Ludwig-Universität Freiburg, Medizinische Fakultät, Institut für Medizinische Psychologie & Medizinische Soziologie, Freiburg, Germany

## Bibliographical details

Christoph Cantzler, Sigrid Harendza

**Gute Besserung – Das Krankenhausspiel**

www.auftragspiel.de

Year of publication: 2020, prize: € 16,90

## Review

Hospital management and the related economic aspects are of central importance for health care and thus crucial for the health and well-being not only of patients but also of all professional groups involved in their treatment and care. However, in undergraduate medical education as well as in the education of other health professions, these topics have so far been rather poorly represented, and they are generally not among the students’ favorites. A more thorough examination of the structural and economic framework conditions of future work and their determinants would be very important, as these have a considerable influence on job satisfaction and the experience of stress in the profession.

It could be the case that sometimes there is just a lack of good ideas on how to present the supposedly dry topic in such a way that, in addition to teaching new insights and skills, it also makes learning fun. This is exactly where help is now available in the form of the “Gute Besserung” hospital game developed by Christoph Cantzler and Sigrid Harendza. In simple terms, the aim is to transfer patients admitted as emergencies to the appropriate ward as quickly as possible and to provide them with adequate medical care so that they are not only satisfied, but also generate revenue for the hospital. It goes without saying that this only works well if resources, i.e. the manpower of nurses and doctors, are used sparingly.

What is difficult in reality is not so easy in the game either. Because, as in real life, different patients present very different challenges: Some have private insurance and therefore are unhappy if they are nevertheless assigned a shared room, while others are infectious and need a single room for this reason. However, their number is naturally limited and for this reason alone, prudent bed management is necessary even "in the smallest hospital in the world". But the competences and possibilities of the medical staff are not available in unlimited quantities either, and so, if the luck of the cards does not play into your hands at the time, you have to ask other players for help if you cannot perform the operation on your own or if the appropriate intravenous drip is not available at the moment.

However, you are also rewarded for good work: the number of smileys grows with increasing patient satisfaction, and good treatment and discharge management increases the number of euros that each player can pile up in front of him or her. But it's not just about maximizing profits for your own department and achieving high patient satisfaction scores. In order to win, you also have to ensure that you do not use up too much of the nurses’ and doctors’ manpower that you were allocated initially. The evaluation of the scores is therefore split into several categories to enable you to refine your skills, for example by optimizing and balancing the various parameters of hospital management through teamwork from round to round.

Even though the overall fun of the game plays the greatest role and you do not need any specific prior knowledge to keep up, the game is still suitable for an entertaining discussion about the rather serious background. The game is therefore not a “serious game” in which you playfully acquire new content, but it can certainly motivate you to acquire it, for example, if you have won a lot of euros but few smileys.

Another plus of the game is that it is almost smock pocket-sized and can therefore be taken anywhere, especially since it does not take up much space when played. As the game only requires a minimum of two players, it is also Coronacompatible; with more players (up to four), however, the level of entertainment and difficulty increases. The board and cards are well-designed and although the rules of the game are not quite trivial – just like hospital management – you can start immediately, because the game instructions work according to the principle of learning by doing. If you are looking for even greater challenges after a few rounds, you can continue playing in expert mode, in which case you can also train your memory skills: in the normal mode you can see which treatment has already been carried out for which patient, but in the expert mode you have to remember this in order to avoid expensive treatment duplication or missing treatments. If that is not enough, you can come up with new rules yourself, which the author team expressly invites you to do. And who has not wanted to manage a hospital according to their own rules for a long time? Definitely worth a go!

## Competing interests

The author declares that he has no competing interests. 

